# Tri-Cyclic Nucleobase Analogs and Their Ribosides as Substrates of Purine-Nucleoside Phosphorylases. II Guanine and Isoguanine Derivatives

**DOI:** 10.3390/molecules24081493

**Published:** 2019-04-16

**Authors:** Alicja Stachelska-Wierzchowska, Jacek Wierzchowski, Michał Górka, Agnieszka Bzowska, Beata Wielgus-Kutrowska

**Affiliations:** 1 Department of Physics and Biophysics, University of Varmia & Masuria in Olsztyn, 4 Oczapowskiego St., 10-719 Olsztyn, Poland; jacek.wie@uwm.edu.pl; 2 Division of Biophysics, Institute of Experimental Physics, University of Warsaw, Pasteura 5, 02-093 Warsaw, Poland; michal.gorka@fuw.edu.pl (M.G.); Agnieszka.Bzowska@fuw.edu.pl (A.B.); Beata.Wielgus-Kutrowska@fuw.edu.pl (B.W.-K.); 3 Biological and Chemical Research Centre, University of Warsaw, Żwirki i Wigury 101, 02-089 Warsaw, Poland

**Keywords:** chemical mutagenesis, fluorescence, nucleobase/nucleoside analogs, purine nucleoside phosphorylase, tautomerism

## Abstract

Etheno-derivatives of guanine, *O*^6^-methylguanine, and isoguanine were prepared and purified using standard methods. The title compounds were examined as potential substrates of purine-nucleoside phosphorylases from various sources in the reverse (synthetic) pathway. It was found that 1,*N*^2^-etheno-guanine and 1,*N*^6^-etheno-isoguanine are excellent substrates for purine-nucleoside phosphorylase (PNP) from *E. coli*, while *O*^6^-methyl-*N*^2^,3-etheno-guanine exhibited moderate activity vs. this enzyme. The latter two compounds displayed intense fluorescence in neutral aqueous medium, and so did the corresponding ribosylation products. By contrast, PNP from calf spleens exhibited only modest activity towards 1,*N*^6^-etheno-isoguanine; the remaining compounds were not ribosylated by this enzyme. The enzymatic ribosylation of 1,*N*^6^-etheno-isoguanine using two forms of calf PNP (wild type and N243D) and *E. coli* PNP (wild type and D204N) gave three different products, which were identified on the basis of NMR analysis and comparison with the product of the isoguanosine reaction with chloroacetic aldehyde, which gave an essentially single compound, identified unequivocally as *N*9-riboside. With the wild-type *E. coli* enzyme as a catalyst, *N*9-β-d- and *N*7-β-d-ribosides are obtained in proportion ~1:3, while calf PNP produced another riboside, tentatively identified as *N*^6^-β-d-riboside. The potential application of various forms of PNP for synthesis of the tri-cyclic nucleoside analogs is discussed.

## 1. Introduction

Some tri-cyclic analogs of the natural purine bases and their glycosides show intense fluorescence, what enables their application as fluorescent probes in the investigations of structure and function of nucleic acids (DNA, RNA) and enzymes related to nucleic acid metabolism or utilizing nucleotide cofactors [1,2]. The most popular probe of this kind is 1,*N*^6^-etheno-adenosine (εAdo), easily produced in the reaction of adenosine with chloroacetic aldehyde (CAA) in aqueous environment.

**Scheme 1:**
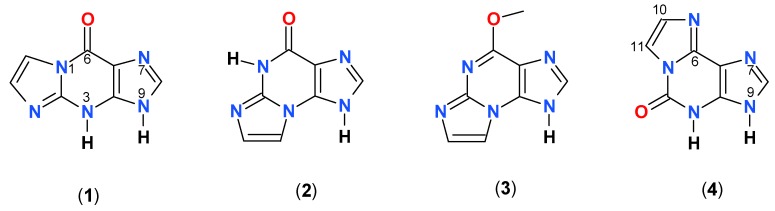
Structures of the nucleobase analogs investigated in this work (from left to right): (**1**) 1,*N*^2^-ethenoguanine, (**2**) *N*^2^,3-ethenoguanine, (**3**) *N*^2^,3-etheno-*O*^6^-methylguanine, (**4**) 1,*N*^6^-ethenoisoguanine. Only one tautomeric form is given for simplicity. Note that the purine numbering is applied (except for the etheno-group atoms).

Other etheno-purine derivatives (see Scheme 1) were also prepared but were reported to display rather discouraging emission properties: This refers to the guanine/guanosine derivatives [3,4,5], as well to the products of the analogous reactions of CAA with 2-aminopurine and its riboside [6,7], and 2,6-diaminopurine [7,8], which were found to be only moderately fluorescent in the neutral aqueous medium. Nevertheless, a thorough spectral examination of those and other etheno-derivatives in various conditions seems to be necessary for further applications.

Tri-cyclic analogs and their ribosides are characterized by moderate biological activity, but some of them reveal promising anti-viral properties [9], recently reviewed by Janz-Wechmann et al. [10,11]. They are known to react with many enzymes of purine metabolism [2,10], and are important intermediates of the chemical mutagenesis [12].

In our laboratory, we are working on an enzyme purine-nucleoside phosphorylase (PNP, E.C. 2.4.2.1), responsible for the regulation of the various nucleosides concentrations within the living cells, and a target of many types of pharmaceutical interventions [13,14]. It is also utilized as a biocatalyst in chemo-enzymatic syntheses of nucleoside analogs of pharmaceutical significance [15,16,17]. Until recently it was presumed that this enzyme is not active towards the tri-cyclic analogs (bases or nucleosides). This conclusion was drawn from the early experiments made in the 80s [2], which however could not take into account a great variety of the molecular forms of this enzyme known today [13,17].

Our introductory investigations have shown that PNP isolated from *E. coli*, which is known to possess a broad specificity toward various base and nucleoside analogs [13], is also active towards tri-cyclic εAdo and its 2-aza analog [18]. The phosphorolytic reaction of εAdo was almost as rapid as other phosphorolytic reactions with natural substrates, like adenosine or guanosine. In the absence of phosphate ions, it is possible to observe the reverse reaction, that is, attachment of the sugar moiety to the tri-cyclic base, where the second substrate is a phosphorylated sugar (in our case α-d-ribose-1-phosphate, R1P). This reaction runs similarly rapidly as the phosphorolytic process.

The purpose of the present work was to examine other similar tri-cyclic nucleobase analogs, in particular guanine and isoguanine derivatives, as potential substrates of analogous reactions, and possibly obtain in this way highly fluorescent compounds, useful for the future research. We have also extended the spectral examination of the above-mentioned etheno-derivatives to include the respective ionic forms, which in some cases are important intermediates in enzymatic catalysis, occasionally identified in enzyme-substrate complexes [19].

## 2. Results and Discussion

### 2.1. Properties of 1,N^2^-ethenoguanine and 1,N^2^-ethenoguanosine

1,*N*^2^-ethenoguanine (1, 5,9-dihydro-9-oximidazo{1,2-a}purine) is one of 2 isomeric etheno derivatives of guanine, its structure similar to one of the known rare t-RNA bases, the so-called Y-base (3,10-dimethyl-1,*N*^2^-ethenoguanine) [3]. 1,*N*^2^-ethenoguanine is a main product of the slow reaction of CAA with guanine [5], and one of three main products of the reaction of DNA with the mutagen vinyl chloride [12,20,21,22]. We decided that more convenient way to obtain this isomer would be a much faster reaction of CAA with 2-amino-6-chloropurine riboside [6], which gave a mixture of etheno-guanine isomers and some ribosides as well, readily separated using semi-preparative high-performance liquid chromatography (HPLC).

1,*N*^2^-ethenoguanine (**1**) was reported to be nonfluorescent in the neutral aqueous medium [3]. Weak emission was observed from its monoanionic form (Table 1), but no significant emission was detected at low pH (~1), where this compound is protonated.

**Figure 1:**
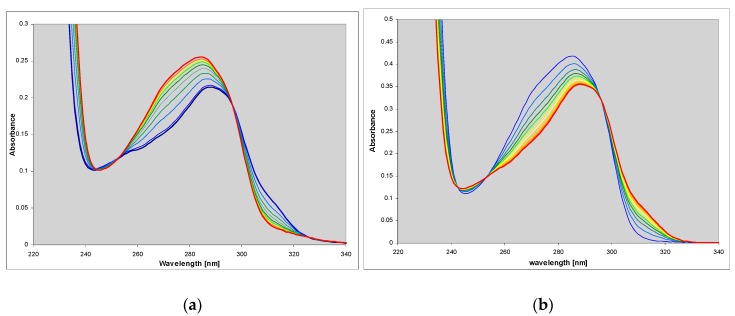
Spectral changes observed during (**a**) the enzymatic ribosylation of 1,*N*^2^-ethenoguanine (1) with 0.5 mM R1P, in HEPES buffer, pH 7.3, catalyzed by purine-nucleoside phosphorylase (PNP) from *E. coli*; (**b**) enzymatic phosphorolysis of 1,*N*^2^-ethenoguanosine by the same enzyme in 50 mM phosphate buffer, pH 7. The time interval is 5 min. Final curves are drawn in red.

We found that this compound is an excellent substrate for PNP from *E. coli* (Figure 1a), with catalytic and Michaelis’ constants comparable to that for ribosylation of the parent guanine (Table 2). HPLC analysis of the reaction mixture has shown that there is one main product (>95%), spectrally and chromatographically identical to the nucleoside generated in the chemical way (Figure 1). The reverse reaction (phosphorolysis of the nucleoside) is also easily observed in the presence of phosphate ions (Figure 1b). These facts may be important in view of a significant mutagenic role of 1,*N*^2^-ethgenoguanine (1) lesion in many organisms, particularly in bacteria [22].

The mutated form of the *E. coli* PNP (D204N) reacted in a qualitatively similar way as the wild-type enzyme, with similar spectral changes and comparable rate.

With the calf enzyme (wild-type or the N243D mutant) only very slow reaction was observed under typical conditions (pH 7.3, 4-(2-hydroxyethyl)-1-piperazineethanesulfonic acid (HEPES) buffer, 25 °C). This reaction was ca. 100-fold slower than the ribosylation of guanine. There was also no marked phosphorolysis of the nucleoside by this enzyme in the phosphate buffer (data not shown). We conclude that PNP probably does not play any substantial role in the process of detoxication of etheno-guanosine in mammals, but this kind of degradation can be important in bacteria.

### 2.2. Spectral Properties of N^2^,3-ethenoguanine 

**Figure 2:**
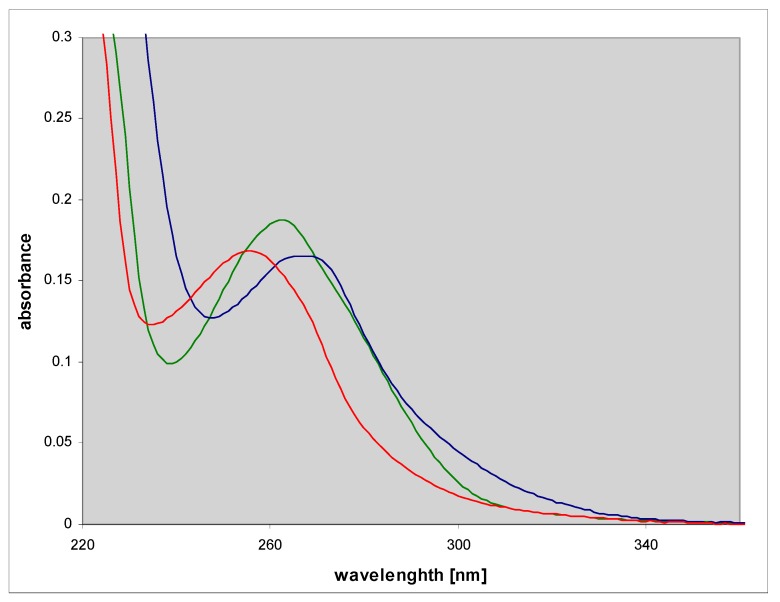
Electronic absorption spectra of the neutral (green), cationic (red) and mono-anionic forms of *N*^2^,3-ethenoguanine (2).

*N*^2^,3-ethenoguanine (**2**), a non-linear isomer of 1,*N*^2^-ethgenoguanine (**1**), is a common and the most persistent lesion in DNA upon treatment with vinyl chloride [12]. Its role in mutagenesis has been addressed in many papers and shown to be significant [23,24,25]. This compound is weakly fluorescent in the neutral aqueous medium and moderately in some organic solvents [3]. The UV absorption of *N*^2^,3-ethenoguanine (**2**) is blue-shifted relative to 1,*N*^2^-ethenoguanine (**1**) (Figure 2), but its acido-basic properties are similar to those of the latter compound (Table 1), however, the ionic forms are nonfluorescent.

We found that *N*^2^,3-ethenoguanine (2) is not a substrate for PNP form *E. coli* and calf spleen. No reaction traces were observed even with 10-fold higher enzyme concentrations than those applied to the synthesis of 1,*N*^2^-ethenoguanosine (see previous section). Ribosides of *N*^2^,3-ethenoguanine are known to be relatively unstable [26,27], and ribose modifications stabilizing the glycosidic bond were used to study the role of this compound in mutagenesis [25]. Probably this instability of the glycoside bond is the main obstacle in its effective enzymatic ribosylation, although it is not clear why other possible ribosylation sites (like *N*7 or *N*^2^) cannot be utilized by the enzymes.

With guanine as a substrate of the ribosylation reaction, we have found moderate, possibly competitive inhibition of the *E. coli* PNP by *N*^2^,3-ethenoguanine (**2**), with estimated K_i_ of 38 μM. This value is not far from the K_m_ value for guanine (ca. 54 μM in our conditions, [13]). We conclude that inability of the enzyme to ribosylate (2) is not due to geometric hindrance, but rather should be ascribed to unfavorable energetic factors, in accordance with previous reports about the relative instability of the glycosidic bond in this compound [23].

### 2.3. N^2^,3-etheno-O^6^-methylguanine *(**3**)*

**Figure 3:**
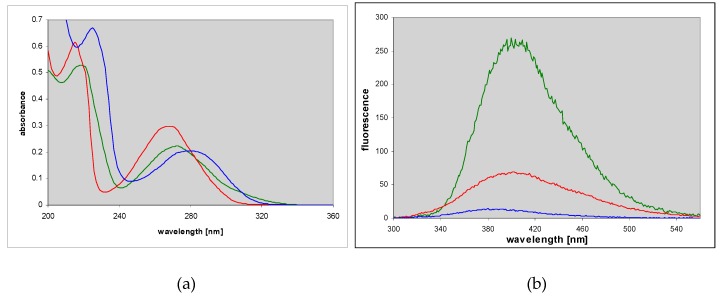
UV absorption (left) and fluorescence spectra (right) of *N*^2^,3-etheno-*O*^6^-methylguanine at various pHs: Cationic form (pH 2, red), neutral (pH 6.5, green) and a monoanionic form (pH 11, blue); excitation at 280 nm.

A known mutagenic guanine derivative, *O*^6^-methylguanine [26,27], reacts readily with CAA to give an essentially single product, identified as *N*^2^,3-etheno-*O*^6^-methylguanine (**3**) [3]. In contrast to the parent guanine derivative, this compound exhibits fairly intense fluorescence in neutral aqueous medium, centered about 400 nm (Figure 3 and Table 1). Its protonated form is also fluorescent, albeit with a lower yield, while the emission from the monoanionic form is only minimal (Figure 3 and Table 1).

**Figure 4:**
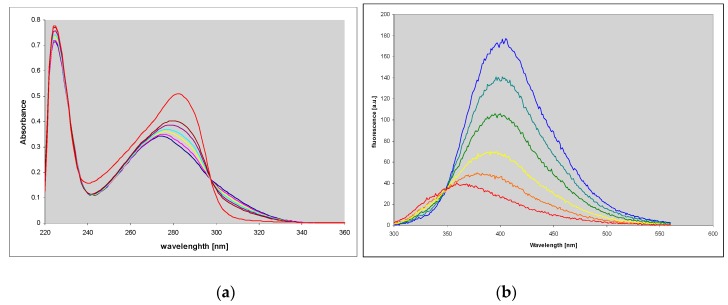
Spectral changes observed during the enzymatic ribosylation of ~35 μM *N*^2^,3-etheno-*O*^6^-methylguanine (**3**) with *E. coli* PNP as a catalyst. (**a**): UV absorption; (**b**): Fluorescence spectra. Conditions: 40 mM HEPES buffer, pH 7.3, 0.5 mM ribose-1-phosphate, 25 °C. The final (red) curves are recorded after ca. 24 h of reaction. Fluorescence excitation was taken at 280 nm.

*N*^2^,3-etheno-*O*^6^-methylguanine (**3**) is also a substrate for PNP from *E. coli*, but the maximal velocity of its ribosylation is rather poor (Table 2). The product riboside spectrally differs from the substrate base, particularly by its weaker fluorescence, so the ribosylation is easy to follow (Figure 3). The reaction progress curve initially seems to be linear (Figure 4b, and Supplementary Materials), pointing to the low K_m_ value. The observed marked differences in the absorption and emission spectra of the product riboside and the parent base is most likely due to prototropic tautomerism (N9H↔N7H) of the latter, not possible for the riboside.

Although the ribosylation rate is slow, the apparent K_m_ value, calculated from the progress curve (see Supplementary Materials), is markedly below 10 μM, so it is somewhat lower than that for the canonical nucleobases [13]. Although the kinetically measured K_m_ cannot be directly used as a measure of binding properties of the purine analog [13], its low value indicates that the geometry of the compound is in this case not unfavorable for the *E. coli* PNP, and the confirms that the main reason for slow ribosylation and the resistance of the parent *N*^2^,3-ethenoguanine (**3**) to the enzymatic ribosylation is probably unfavorable energetics.

The ribosylation reaction is fully reversible by the addition of a phosphate buffer up to a concentration of ~5 mM (even after 24 h, data not shown). We did not attempt to isolate the pure riboside due to the low efficiency of the synthetic process, but it would be interesting to check the ribosylation site, which may be different from the “canonical” *N*9, explaining spectral differences between this product and the parent base.

*N*^2^,3-etheno-*O*^6^-methylguanine (**3**) effectively competes with guanine in the ribosylation process, acting as quasi inhibitor of the *E. coli* PNP, with estimated K_iapp_ value 7.5 ± 0.8 μM (competitive model; average of 3 determinations), in agreement with the K_m_ value, estimated from kinetic data (see the previous paragraph). With the calf enzyme, no such inhibition is observed under similar conditions.

### 2.4. 1,N^6^-etheno-isoguanine (**4**)

Isoguanine (2-hydroxy-6-aminopurine, isoGua) is a guanine isomer, found in several living species [28], and it is also a product of adenine reaction with the hydroxyl radical, generated radiochemically [29]. Isoguanine and isoguanosine is characterized by the readily detectable, solvent-induced keto-enol tautomerism [28], and ambiguity of coding properties when incorporated into DNA/RNA [30].

The nucleoside of isoGua, isoguanosine, is rather slowly phosphorolysed by the *E. coli* PNP (V_max_ ca. 40-fold lower than that for guanosine, data not shown), and we did not see any such reaction catalyzed by the calf enzyme, in accordance with the general rule [13], that 6-aminopurine ribosides, not possessing mobile proton at the *N*1 position, do not react with trimeric forms of PNP, including mammalian enzymes.

Isoguanine reacts readily with CAA to produce a single, and highly fluorescent product, 1,*N*^6^-etheno-isoguanine (εisoGua, (4)). The etheno-isoguanine fluorescence was long ago proposed as a tool for analytical quantitation of 9-β-d-arabinofuranosyl-2-fluoroadenine (fludarabine, [31]), but, to our knowledge, no systematic examination of the emission properties of **ε**isoGua (4) exists in the literature. Basic spectral data for this compound are given in Table 1.

**Figure 5:**
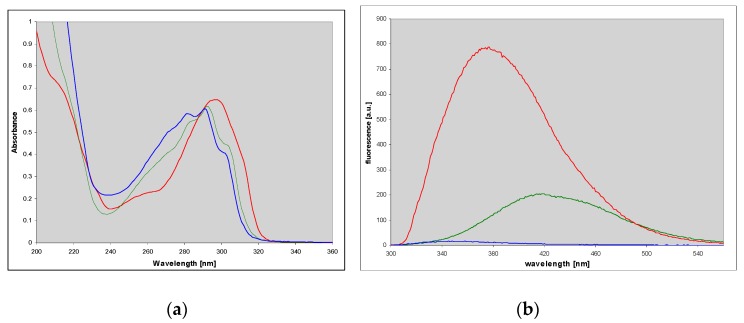
Electronic absorption (**a**) and fluorescence (**b**) spectra of 1,*N*^6^-etheno-isoguanine (**4**) in aqueous medium at various pH: Cationic form (pH 2, red), neutral (pH 6.5, green) and a monoanionic form (pH 11, blue). Fluorescence excitation was taken at 290 nm.

Spectrophotometric titrations of εisoGua (4) (for details, see the Supplementary Materials) indicate that this compound exists as a neutral species at pH 4.5–7, and above pH 8 undergoes deprotonation, and there is probably a second deprotonation at pH ~ 13. The anionic forms are virtually nonfluorescent, while the neutral form and the cation are strongly fluorescent (Figure 5 and Table 1). Fluorimetric titrations give results essentially in agreement with the UV data (not shown).

**Scheme 2:**
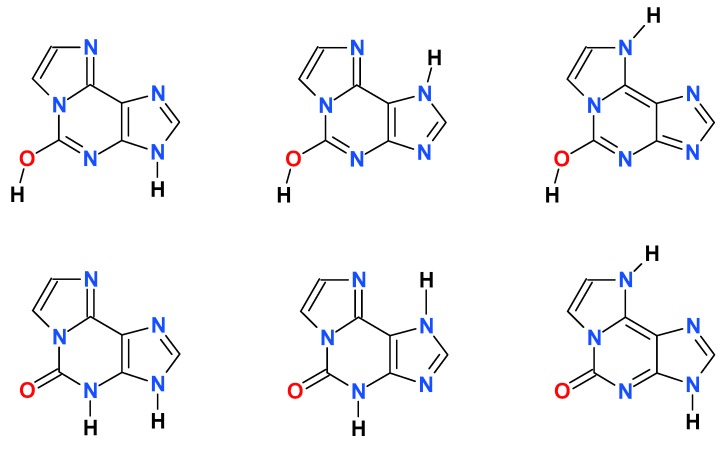
Some of the possible tautomeric forms of 1,*N*^6^-etheno-isoguanine (**4**).

**Figure 6:**
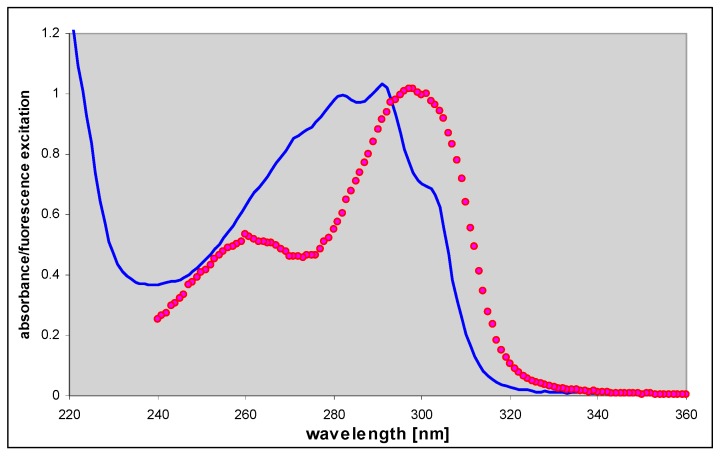
Comparison of the UV absorption (blue, solid line) and corrected fluorescence excitation (red points) spectrum of 1,*N*^6^-etheno-isoguanine (**4**), measured at pH 6.5. The fluorescence excitation spectrum was measured with observation at 415 nm and resolution 2.5 nm.

Fluorescence properties of εisoGua (4) strongly suggest a ground-state tautomeric equilibrium. Fluorescence decay, measured at excitation 280 nm at pH 6.5 (neutral form), is clearly nonexponential, but well approximated by a bi-exponential decay function (data not shown), with decay times of 5.1 and 3.4 ns (see Table 1). Similar situations exist in 1,*N*^6^-adenine, but not its nucleoside [32]. Moreover, the fluorescence excitation spectrum of the neutral form of (4) at pH 6.5, although virtually observation-independent (data not shown), is clearly distinct from the UV absorption (Figure 6 below), suggesting the existence of a third not-emitting or weakly emitting species. The tautomeric equillibria in εisoGua (4) are even more complex that those of etheno-adenine, where only a *N*(9)H—*N*(7)H-*N*^6^H prototropic tautomerism is likely [18], while in (4) the situation is additionally complicated by possible keto-enol tautomerism (see Scheme 2, below). This problem requires therefore further elucidation. 

**Figure 7:**
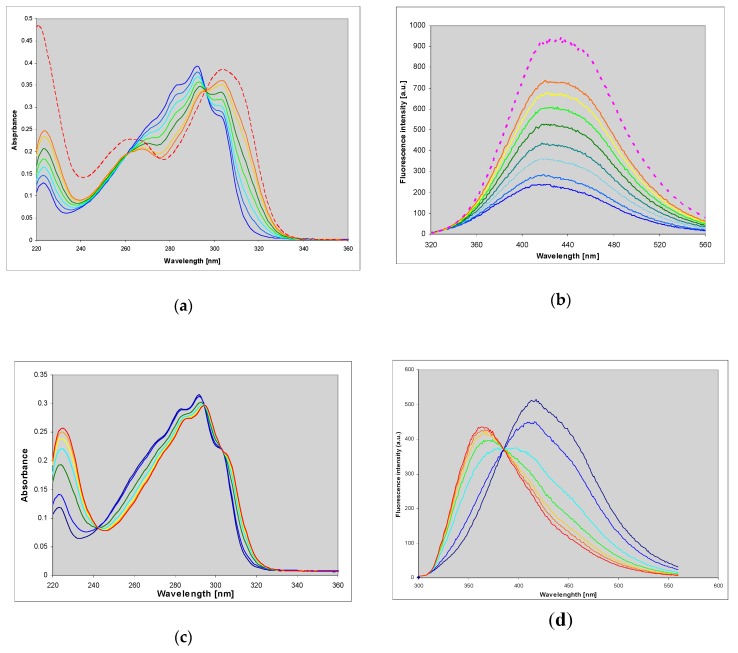
Changes in UV absorption (**a**,**c**) and fluorescence (**b**,**d**) spectra during the enzymatic ribosylation of εisoGua (**4**) with calf (**a**,**b**) and *E. coli* (**c**,**d**) PNP as catalysts. Excitation was at 290 nm. Final curves are colored in red. The reactions were carried out in 40 mM HEPES, pH 7.3, at 25 °C, with 0.5 mM α-d-ribose-1-phosphate as a ribosyl donor. Initial substrate concentrations in fluorescence measurements were 10–25 μM.

**Figure 8:**
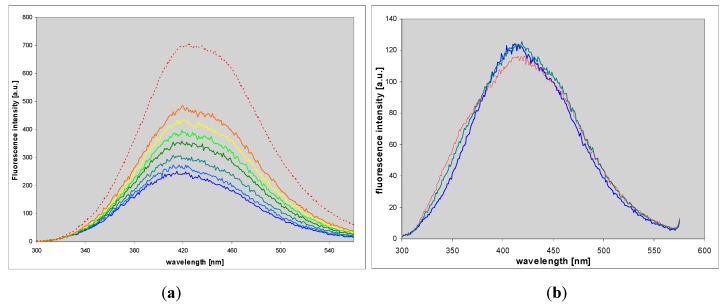
Changes in the fluorescence spectrum during the enzymatic ribosylation of 1,*N*^6^-etheno-isoguanine (4) by the mutated forms of PNP: (**a**), the N243D mutant of the calf PNP; (**b**): The D204N mutant of the *E. coli* enzyme. Final curves are colored in red. Initial substrate concentration was ca. 10 μM. Other reaction conditions were identical as on Figure 7. Excitation was taken at 290 nm.

**Figure 9:**
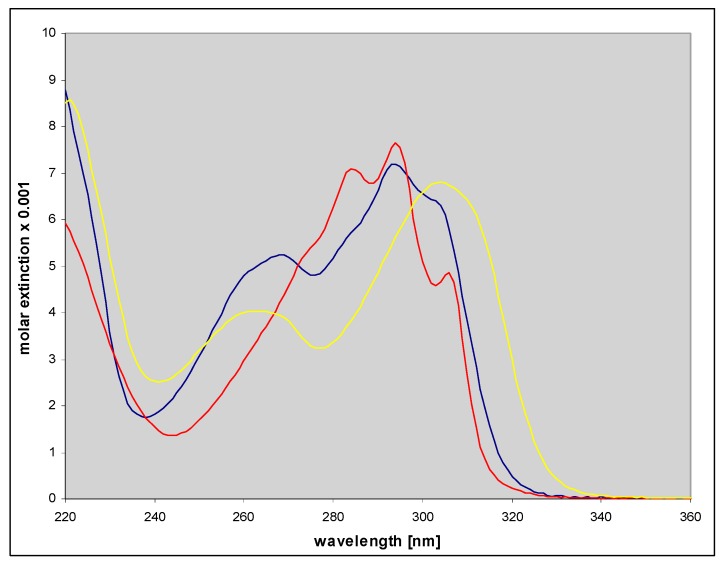
Electronic absorption spectra of three ribosides of 1,*N*^6^-etheno-isoguanine, purified by HPLC. Spectra of *N*9- (red color) and *N*7-β-d-riboside (blue) were measured in 50 mM phosphate buffer, pH 6.5, and that of *N*^6^-riboside (yellow), at pH 7.

1,*N*^6^-etheno-isoguanine (**4**) is a moderately good substrate for PNP from various sources, but reactions with various types of the enzyme vary not only in rates but also in products obtained, giving at least 3 types of ribosides (Figure 7, Figure 8 and Figure 9). This situation is somewhat reminiscent of the ribosylation of 1,*N*^6^-ethenoadenine, where also 3 ribosides were obtained [14,18], all of them fluorescent. In the case of (**4**), all three ribosylated species are also fluorescent, although with different yields and decay times (Table 1).

#### Ribosides of 1,*N*^6^-etheno-isoguanine 

**Scheme 3:**
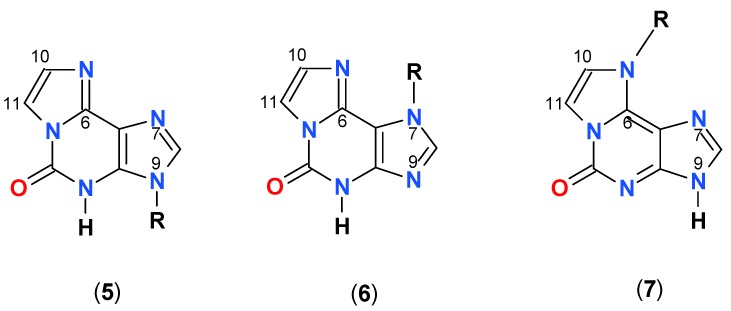
Structures of the obtained ribosides of 1,*N*^6^-etheno-isoguanine (**4**). R=β-d-ribosyl: (**5**) 1,*N*^6^-etheno-*N*9-β-d-ribosylisoguanosine; (**6**) 1,*N*^6^-etheno-*N*7-β-d-ribosylisoguanosine; 1,*N*^6^-etheno-*N*^6^-β-d-ribosylisoguanosine (**7**).

As mentioned above, three types of ribosides of 1,*N*^6^-etheno-isoguanine (**4**) can be generated. One chemically from isoguanosine reacting with CAA, and all three enzymatically, using various types of PNP as a biocatalyst (Scheme 3). The reaction of isoguanosine with CAA was fairly rapid at pH 4.5 and room temperature and gave essentially a single product, identified as *N*9-β-d-riboside (5) of εisoGua. Enzymatic ribosylation gave an additional 2 products, clearly not identical with (5). Ribosylation catalyzed by the enzyme from *E. coli* gave two products: The highly fluorescent N9-riboside (5, minor product), and another riboside with less intense fluorescence shifted to ~355 nm (Figure 7 below, lower panels). The analogous reaction catalyzed by the calf PNP gave one main product, very intensely fluorescent, but with UV absorption spectrum markedly shifted to the longer wavelengths (Figure 7, upper panels). The observed ambiguity of the ribosylation sites is analogous to that described earlier for the enzymatic ribosylation of etheno-adenine [18] and some 8-azapurines [33].

NMR spectral analysis was necessary to identify the ribosylation sites (Scheme 3). Identification of the three ribosides of εisoGua (4) was based mainly on connectivities observed in ^1^H, ^13^C HMBC spectra. ^1^H, ^1^H COSY spectra allowed for the assignment of all aliphatic sugar protons. Of the three signals of aromatic base protons, the two protons of the etheno group were identified due to the scalar coupling between them, with the εisoGua (4) H8 proton identified by elimination. The presence of C8-H1’ and C1’- H8 correlations in the HMBC spectrum allowed for the identification of two of three samples as either *N*7 (6) or *N*9- (5) ribosides. The former riboside (6) was identified by the presence of C6- H1’ and C6-H8 correlations, where C6 was assigned based on its relatively low chemical shift (typical values and the substituent effects have been reviewed in Reference [34]), compared to the expected chemical shift of C4. By elimination, the remaining sample was identified as the *N*9-riboside (5), in agreement with chemo-enzymatic identification provided in the previous paragraph. The *N*^6^-riboside (7) was identified by the presence of the correlation of both etheno protons with C1’ and (in the ^1^H, ^15^*N* spectrum) *N*^6^. Selected chemical shifts are given in Table 3, with the full list provided in Supplementary Materials.

For (5) only one of the etheno protons is observable in 1H, 13C spectra, possibly due to (slow or intermediate) chemical exchange. 

In the 50 mM phosphate buffer, pH 6.5, the *N*9-riboside (5) is not phosphorolysed by the calf PNP, but the reaction with the *E. coli* enzyme is fairly rapid (see Supplementary Materials). Even more rapid is phosphorolysis of the *N*7-riboside (6) by the same type of PNP, while the *N*^6^-riboside (**7**) is only slowly deribosylated by the calf enzyme, but much more rapidly by the *E. coli* PNP (data not shown). This is to be compared with 1,*N*^6^-ethenoadenosine, resistant to the calf enzyme, but rapidly phosphorolysed by the *E. coli* PNP [18]. All three ribosides are fairly stable in neutral aqueous environment, and no significant instability was seen at pH 11 (data not shown). The respective emission yields of the neutral forms are comparable to those of the best-known fluorescent probes [1,18,33,36]. For these reasons, they seem to be more universal as probes than the somewhat unstable 1,*N*^6^-ethenoadenosine [1].

Various types of PNP mutated at the active site are known, some of them with qualitatively different catalytic characteristics [33,37,38]. We have therefore checked two of such mutants, namely the D204N mutant of the *E. coli* PNP and the “complementing” N243D mutant of the calf enzyme. Both mutations were previously shown to alter ribosylation specificity of PNP with 6-amino-purines [37] and 8-azapurines [33,38] as substrates.

In the case of εisoGua (4), the mutation effect is not very pronounced, with the exception of the *E. coli* PNP, where emission spectrum of the product mixture was shifted to the red, relative to the wild-type PNP reaction (Figure 7 and Figure 8). This latter fact suggests a larger contribution by the *N*9-riboside (5) as a ribosylation product, with a possible contribution by other product(s). Reaction rates (catalytic constants) were comparable to those obtained for the wild-type enzymes. These results are analogous to those reported for 1,*N*^6^-ethenoadenine as a substrate [18].

## 3. Experimental Section

Isoguanine, isoguanosine, and their derivatives were obtained from Dr. Jerzy Sepioł (Polish Academy of Sciences). Chloroacetaldehyde, 7-methylguanosine, *O*^6^-methylguanine and 2-amino-6-chloropurine riboside were from Sigma-Aldrich. The ribose source for enzymatic ribosylation, α-d-ribose-1-phosphate (R1P) has been prepared as 100 mM solution as previously described [18] and kept frozen.

**Figure 10:**
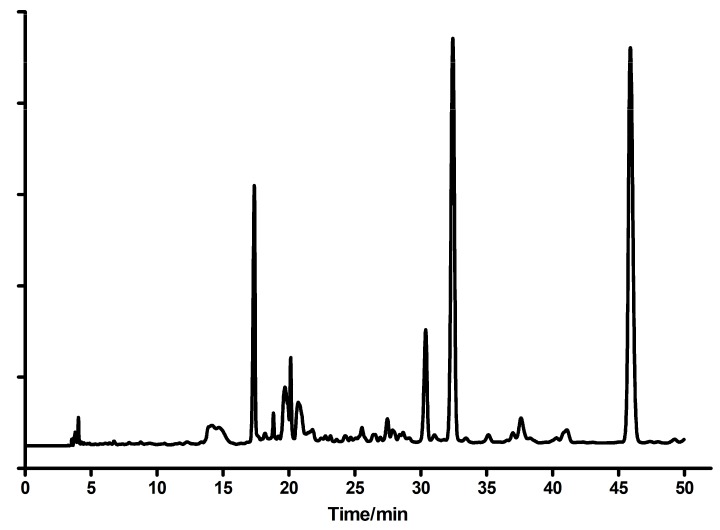
The HPLC elution profile of the post-reaction mixture (2-amino-6-chloropurine riboside reacting with chloroacetaldehyde). Based on UV and fluorescence spectra, the peak at 17 min was identified as guanine, at 30 min as *N*^2^,3-ethenoguanine (**2**), at 32 min as 1,*N*^2^-ethenoguanine (**1**), and that of 46 min as 1,*N*^2^-ethenoguanosine. A small amount of the starting substance, 2-amino-6-chloropurine riboside, is not envisioned (retention time >50 min).

Etheno-derivatives of guanine and guanosine were obtained analogously to Hořejši et al. [6], from the reaction of 2-amino-6-chloropurine riboside with ca 7-fold molar excess of chloroacetaldehyde (CAA, [5,6]). The reaction was carried out in aqueous environment, at room temperature and pH 4-4.5, controlled by addition of sodium bicarbonate. After 3 days, the reaction mixture was acidified to pH ~1 and boiled by ~3 min. HPLC analysis (Figure 10) revealed a significant degree of hydrolysis of chloride, as well as of ribosyl moiety.

The main product was identified as 1,*N*^2^-ethenoguanine (1) on the basis of UV spectra in various conditions and lack of fluorescence. The second isomer, *N*^2^,3-ethenoguanine (2), was identified on the basis of its fluorescence and UV spectra shifted to ~260 nm. The third major product was 1,*N*^2^-ethenoguanosine, which was proved by its UV absorption and ready enzymatic phosphorolysis to 1,*N*^2^-ethenoguanine (1, see Section 2.1). Smaller amounts of guanine and the starting 2-amino-6-chloropurine riboside were also found. The main products were purified via the semi-preparative HPLC (C-18 column), eluted with water (10–12 min), followed by water-methanol gradient (10–30%, 40 min). The analogous method, with various reaction times, was used for other compounds (see below). The progress of the reactions was monitored spectrally or, wherever possible, also fluorimetrically.

*N*^2^,3-etheno-*O*^6^-methylguanine (**3**) was obtained from the reaction of aqueous CAA with *O*^6^-methylguanine under weakly acidic conditions (ca. 3 days). There was essentially one product, easily crystallized after the neutralization of the reaction mixture. Demethylation of *N*^2^,3-etheno-*O*^6^-methylguanine (**3**) in boiling 1 N HCl [3] led to *N*^2^,3-ethenoguanine (**2**), identical to the previously obtained sample (see above).

Etheno derivatives of isoguanine (2-hydroxy-6-aminopurine) and isoguanosine were prepared in analogous ways, but the nucleoside was somewhat more reactive (reaction time was 2–3 days) then the base (7 days). In both cases only a single product, easily identified as the 1,*N*^6^-etheno derivative, was obtained and purified using semi-preparative HPLC with methanol gradient as the main eluent (see Supplementary Materials).

The detailed procedure for (**4**): Isoguanine (200 mg) dissolved initially in ~10 mL of 1 M HCl, added 1 mL of aqueous CAA, reaction pH elevated to ~2 by addition of bicarbonate, and left overnight. The next day, the reaction pH elevated to ~3, and after 24 h to 4-4.5 and was maintained by bicarbonate and/or acetic acid. After 7 days the crude precipitate was collected and dried. HPLC analysis showed ~70% progress of the reaction. The crude precipitate was used as such for the ribosylation experiments and aliquot purified using HPLC.

**Figure 11:**
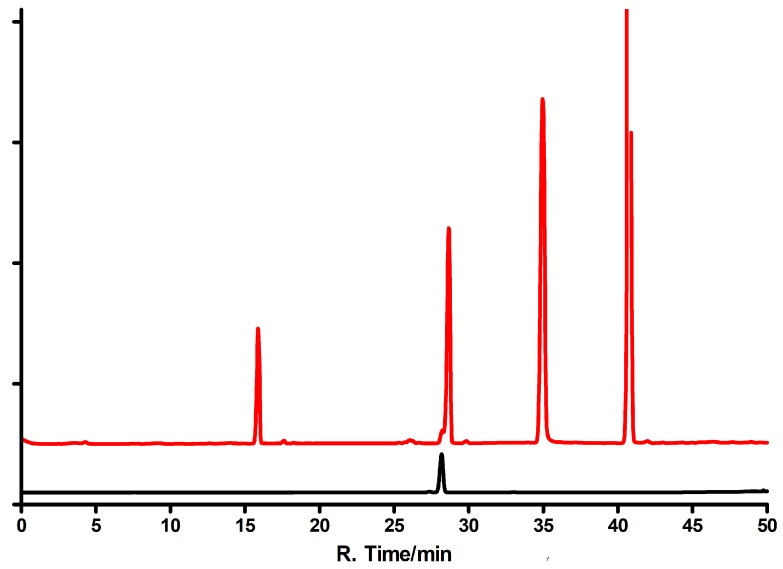
HPLC elution profiles of the reaction mixtures resulting from enzymatic syntheses of the ribosides of 1,*N*^6^-ethenoisogusanine (**4**). Upper curve: The reaction catalyzed by the *E. coli* PNP; lower curve: The reaction catalyzed by calf PNP. The peak with the retention time of 15 min is isoguanine; 28 min indicates either *N*9-riboside (5, upper curve) or *N*^6^-riboside (7, lower curve); ~35 min indicates substrate (**4**), and ~44 min is the *N*7 riboside (6). Elution program: 0–15 min water, 15–45 min methanol gradient 10–30%.

General procedure for milligram-scale enzymatic syntheses of εisoGua (4) ribosides is as follows: 10 mg of substrate dissolved in diluted aqueous ammonia (ca. 10 mL) and stepwise added to ca. 5 mL of 100 mM HEPES buffer, pH~7, containing ca. 10 mM solution of R1P and 20 µL of concentrated (~2.6 mg/mL) E. coli PNP. The reaction was carried out at 30 °C for 3 days, the mixture concentrated, and products separated as described below. Typical HPLC profiles are shown on Figure 11.

Product separation and purification used HPLC on a UFLC system from Shimadzu (Kyoto, Japan) equipped with UV (diode-array) detection at 260, 280 and 315 nm, and a fluorescence detector. The column used was a Kromasil reversed-phase a semi-preparative C-18 column (250 × 10 mm, 5-μm particle size). Elution was initially (10–15 min) isocratic, followed by water-methanol gradient (usually 10–30% methanol for 35–40 min).

For kinetic analyses, enzymatic ribosylation reactions were carried out in 1 mL cuvettes (pathlength 4 mm) in ~50 mM HEPES buffer, pH 7.3, using ca. 0.5 mM R1P as a ribose source. On a larger scale, reactions were run in Eppendorf tubes, volume 2–3 mL, using either R1P or 7-methylguanosine as ribose sources. Phosphorolysis reactions were run in 50 mM phosphate buffer, pH 7.0 or 6.5. Data were analyzed by standard methods (see Supplementary Materials).

Fluorescence spectra were measured on a Varian Eclipse instrument (Varian Corp., Palo Alto, CA, USA), and UV absorption kinetic experiments were performed on a Cary 5000 (Varian) thermostated spectrophotometer. Fluorescence yields were determined relative to tryptophan (0.15) or 1,*N*^6^-ethenoadenosine in water (0.56, [2]). Spectra were measured in semi-micro cuvettes, pathlength 4 mm, to diminish the inner-filter effect. Fluorescence decay was measured and analyzed using a FluoTime 200 lifetime fluorometer (PicoQuant GmbH, Germany), equipped with an R3809U-50 microchannel-plate photomultiplier (MCP-PMT, Hamamatsu, Japan), with 280 nm excitation by sub-nanosecond pulsed LED, as previously described [39]. All buffers were of analytical grade and show no fluorescence background. 

NMR measurements were performed on a Bruker Avance III HD 800 MHz spectrometer equipped with a cryogenically-cooled H-C/N-D TCI probe at 25 °C (sample identified as N^7^riboside) or 50 °C (the other two samples). For all samples the following spectra were acquired: A standard proton spectrum, a ^1^H, ^1^H gradient selected COSY, a gradient selected ^1^H, ^13^C HSQC and a gradient selected ^1^H, ^13^C HMBC tuned for 7 Hz J coupling with a double low pass J filter. For the sampled identified as the *N*7-riboside a gradient selected ^1^H, the ^15^*N* HMBC experiment tuned for 15 Hz J coupling was also acquired.

^1^H chemical shift was referenced by the field-locked substitution method using a <1% sample of TMS in DMSO-d6, ^13^C and ^15^*N* chemical shifts were referenced using the unified chemical shift scale [40]. 2D spectra were processed using the TopSpin 3.5 pl7 software package (*Bruker*) and inspected using the Sparky program [41] with manual peak-picking.

Recombinant *E. coli* and calf spleen PNPs as well as their mutated forms were expressed in *E. coli* and purified according to the procedures described earlier [42,43].

## 4. Conclusions

A known chemical mutagen and carcinogen, vinyl chloride, acts as a modifier of nucleobases, in particular, adenine and guanine moieties [12], which upon extending the heterocyclic system change the respective coding properties [21,25,44]. We have found that at least in bacteria the modified nucleosides can be further degraded by the bacterial PNP, the fact that may account for bacteria’s resistance to mutagenesis, together with the confirmed role of the bacterial glycosylases [45] and deoxyribosyl-transferases [45,46] in this process. PNP may have also some role in the metabolism of the rare t-RNA component, the Y-base and its nucleoside, wyosine.

Another important application of PNP is in chemo-enzymatic synthesis of bioactive nucleoside analogs, utilizing, among others, various types of PNP [14,16,17,46,47]. This application may be extended to tri-cyclic nucleobase analogs, particularly to adenine, isoguanine, and guanine derivatives. As shown in this and the previous [18] works, various forms of PNP are able to synthesize ribosides of such extended nucleobase analogs. In particular, *N*9-β-d-riboside of (1) can be obtained quantitatively from (1), and *N*9-β-d- and *N*7-β-d-ribosides of (4) as a mixture, using the *E. coli* PNP as a biocatalyst. Additionally, a non-typical *N*^6^-β-d-riboside (7) of εisoGua (4) can be nearly quantitatively obtained from (4) using the calf enzyme.

The next work (in preparation) shows that also various etheno-derivatives of the amino-purines, including etheno-2-aminopurine isomers, readily react with PNP to give various ribosides. The above results indicate need for further investigations on the enzymatic ribosylation of various nucleobase analogs to improve specificity and yields. Since the number of the known molecular forms of PNP (natural and mutated) is large [13,16], there is a good chance to obtain specific and efficient biocatalysts for a given task.

Finally, attention should be drawn to the new, highly fluorescent nucleoside analogs, derived from mutagenic *O*^6^-methylguanine and isoguanine. These compounds may be good candidates for the study of ligand-binding equillibria using various PNP forms and for analytical applications.

## Figures and Tables

**Table 1:** Ionization constants (pK_a_ values) and spectral parameters of neutral and ionic forms* of the investigated compounds. The UV spectral data are compiled from refs [1,2,3], and checked in this work, and fluorescence parameters determined in this work, except those for *N*^2^,3-ethenoguanine.

Compound	pK_a_ ±0.1	Form* (pH)	UV Absorption		Fluorescence		
			λ_max_ {nm}	ε_max_ {M^−1^cm^−1^}	λ_max_ {nm}	ϕ	τ {ns}
1,*N*^2^-ethenoguanine	~2 ~9.5	n (6) c (1) ma (11)	287 291 303	9700 ~8600 7200	- - ~400	- - <0.01	- - nd **
*N*9-β-d-ribofuranosyl-	8.8	n (6.5) c (1) ma (11)	284 nd 307	11900 nd 8400	- - ~400	- - <0.01	- - nd
*N^2^*,3-ethenoguanine	~2.5; 8.8	n (6.5) ma (11) c (1)	261 266 255	12850 11300 11500	400 - -	0.02 - -	1.38 - -
*N*^2^,3-etheno- *O*^6^methylguanine	~3.5; ~9	n (6.5) c (2) ma (12)	272 268 280	9900 13300 9100	405 395 ~380	0.11 0.035 <0.01	nd nd nd
1,*N*^6^-etheno- isoguanine	3.5; 8.0	n (6.5) c (2) ma (11)	292 296 290	7000 7300 6900	415 385 350	0.17 0.44 ~0.01	5.15; 3.4 nd nd
*N*9-β-d-ribofuranosyl-	2.4; 7.1	n (5.5) c (1.3) ma (11)	295 294 281	7200 ~7000 ~7400	415 380 -	0.34 0.275 <0.01	6.1 4.4; 0.81 nd
*N*7-β-d-ribofuranosyl-	2.8; ~8.5	n (6) c (2) ma (9)	294 301 297	7100 ~7200 ~6500	360 355 365	0.036 ~0.4 ~0.2	0.8; 5.2 3.5 nd
*N*^6^-β-d-ribofuranosyl-	~3; ~8.5	n (6.5) c (1.7) ma (11)	303 298 312	6900 nd nd	425 405 450	0.66 0.82 ~0.03	nd nd nd

* n—neutral form; c—cation; ma—monoanion; ** nd—not determined.

**Table 2:** Kinetic parameters for the enzymatic ribosylation of nucleobase analogs investigated in this work, in 40 mM HEPES buffer, pH 7, by α-d-ribose-1-phosphate, using various forms of PNP (wt = wild type). Standard errors are estimated to be ~15%.

Substrate/Enzyme	K_m_ (µM)	V_max_ (relative) *	Approximate Product Ratio: *N*9: *N*7-riboside: *N*^6^-riboside
1,*N*^2^-ethenoguanine /calf PNP-wt**	nd	<0.5	predominantly *N*9
1,*N*^2^-ethenoguanine /calf PNP-mutantN243D	nd	-	-
1,*N*^2^-ethenoguanine /*E.coli* PNP-wt	48	18	predominantly *N*9
1,*N*^2^-ethenoguanine /*E.coli* PNP-mutantD204N	nd	>15	predominantly *N*9
*N*^2^,3-ethenoguanine /calf PNP-wt	-	<0.1	-
*N*^2^,3-ethenoguanine /*E.coli* PNP-wt	-	<0.1	-
*N*^2^,3-etheno-*O*^6^-methylguanine /calf PNP-wt	-	<0.1	-
*N*^2^,3-etheno-*O*^6^-methylguanine /*E.coli* PNP-wt	~6	~0.3	Nd
1,*N*^6^-ethenoisoguanine /calf PNP-wt	~160	1.6	1:1:10
1,*N*^6^-ethenoisoguanine /calf PNP- mutantN243D	nd	~1	predominantly *N*^6^
1,*N*^6^-ethenoisoguanine /*E.coli* PNP-wt	98	~24	1:4:0.1
1,*N*^6^-ethenoisoguanine /*E.coli* PNP-mutantD204N	nd	>5	5:5:0

* relative to V_max_ of guanine ribosylation under the same conditions (=100); error ~10%; ** wt = wild type.

**Table 3:** Selected chemical shifts of the three ribosides of εisoGua. Chemical shift labels follow the naming convention of IUPAC-IUBMB-IUPAB [35], extended for the etheno protons (see Scheme 1). NA – resonance not assigned. For the *N*7- and *N*9-ribosides, atoms in the positions 10 and 11 could not be unequivocally differentiated, and the two possible values are slash-separated.

Atom	Sample/Chemical Shift (PPM)		
	*N*^6^-riboside	*N*7-riboside	*N*9-riboside
H1’	6.511	5.987	5.712
H8	7.822	8.262	7.753
H10 *	7.775	7.301/7.79	7.212/7.520
H11 **	7.827		
C1’	90.293	89.833	89.114
C5	NA	104.600	NA
C6	138.682	137.918	141.788
C8	137.799	140.026	135.525
C10	113.386	130.645/113.699	NA/111.558
C11	116.951		

* H7 or C7 according to IUPAC notation; ** H8 or C8 according to IUPAC notation.

## Supplementary Materials

The following are available online, Figure S1: Time-dependence of fluorescence intensity (blue: at 400 nm; yellow: at 460 nm) measured during the ribosylation of *N*^2^,3-etheno-*O*^6^-methylguanine (35 μM) with R1P (0.5 mM) as a ribosyl donor, with *E. coli* PNP as a catalyst., at pH 7.3 and temperature 25 °C. In red color, solid line: a theoretically calculated progress curve, assuming K_m_ = 7 μM and Michaelis’-Menten kinetics. Fluorescence excitation was at 290 nm. Figure S2: Spectrophotometric titrations of 1,*N*^6^-etheno-isoguanine. Left: determination of the lower (basic) pKa value: pH values from 2.9 (red) to 5.5 (violet); Right: determination of the upper (acidic) pK value: pH from 6.25 (red) to 11.5 (violet). The fitted pK_a_ values: 3.5 and 8.1 (±0.2). Figure S3: Double-reciprocal (Michaelis’-Menten) plots for ribosylation of 1,*N*^2^-ethenoguanine (right) and 1,*N*^6^-ethenoisoguanine (right), catalyzed by the *E. coli* PNP, wild-type. The obtained values of K_m_ were 48 μM and 98 μM, respectively. Conditions: 50 mM HEPS buffer, pH 7.3, with R1P (0.5 mM) as a ribosyl donor, temperature 25 °C. Figure S4: Phosphorolysis of *N*7- (left) and *N*9-β-d- (right) ribosides of 1,*N*^6^-etheno-isoguanosine in 50 mM phosphate buffer, pH 6.5, at 25 °C, catalyzed by the *E. coli* PNP. Table S1. Assigned chemical shifts of the three ribosides of εisoGua in DMSO-d6 at 25 °C (*N*7-riboside) or 50 °C (*N*^6^ and *N*9-ribosides). Chemical shift labels follow the naming convention of [42], extended for the etheno protons (see Scheme 1). NA—resonance not assigned. For the *N*7 and *N*9-ribosides atoms in the positions 10 and 11 could not be unequivocally assigned and the two possible values are slash-separated.

## Abbreviations

CAA; chloroacetic aldehyde; COSY; Correlation spectroscopy; DMSO-d6; Hexadeuterodimethyl sulfoxide; εAdo; 1,*N*^6^-ethenoadenosine; εisoGua: 1,*N*^6^-ethenoisoguanine; HMBC; Heteronuclear multiple-bond correlation; HPLC; high-performance liquid chromatography; HSQC; Heteronuclear single-quantum correlation; Isoguanine; isoGua; 2-hydroxy-6-aminopurine; Isoguanosine; *N*9-β-d-ribosyl- of the foregoing; NMR; Nuclear magnetic resonance; R1P: α-d-ribose-1-phoshate; TMS; tetramethylsilane.
