# Supplementary material for: Tri-Cyclic Nucleobase Analogs and Their Ribosides as Substrates of Purine-Nucleoside Phosphorylases. II Guanine and Isoguanine Derivatives
Source: Molecules. 2019 Apr 16;24(8):1493. doi: 10.3390/molecules24081493 (PMC6514686; doi:10.3390/molecules24081493)
Supplement: Supplementary file 1 [file molecules-24-01493-s001.pdf]

Supplementary Materials

Table S1. Assigned chemical shifts of the three ribosides of εisoGua in DMSO-d6 at 25° C (*N*7-riboside) or 50° C (*N*^{6} and *N*9-ribosides). Chemical shift labels follow the naming convention of [41], extended for the etheno protons (see Scheme I.). NA – resonance not assigned. For the *N*7 and *N*9-ribosides atoms in the positions 10 and 11 could not be unequivocally assigned and the two possible values are slash-separated.

Atom	Sample/Chemical Shift [PPM] *N*^{6}-riboside	*N*7-riboside	*N*9-riboside
H1'	6.511	5.987	5.712
H2'	4.408	4.556	4.625
H3'	4.152	4.167	4.125
H4'	4.003	3.989	3.962
H5' / H5''	3.607/3.647	3.571/ 3.719	3.548/ 3.661
OH2'	5.405	5.414	NA
OH3'	5.099	5.197	NA
H8	7.822	8.262	7.753
H10*	7.775	7.301/7.79	7.212/7.520
H11**	7.827		
C1'	90.293	89.833	89.114
C2'	75.180	74.114	73.484
C3'	70.756	70.175	71.497
C4	86.658	86.697	86.595
C5'	61.727	61.817	62.463
C5	NA	104.600	NA
C6	138.682	137.918	141.788
C8	137.799	140.026	135.525
C10*	113.386	130.645/113.699	NA/111.558
C11**	116.951		
N1	183.031	NA	NA
N6	157.149	NA	NA

*H7 or C7 according to IUPAC notation; **H8 or C8 according to IUPAC notation.

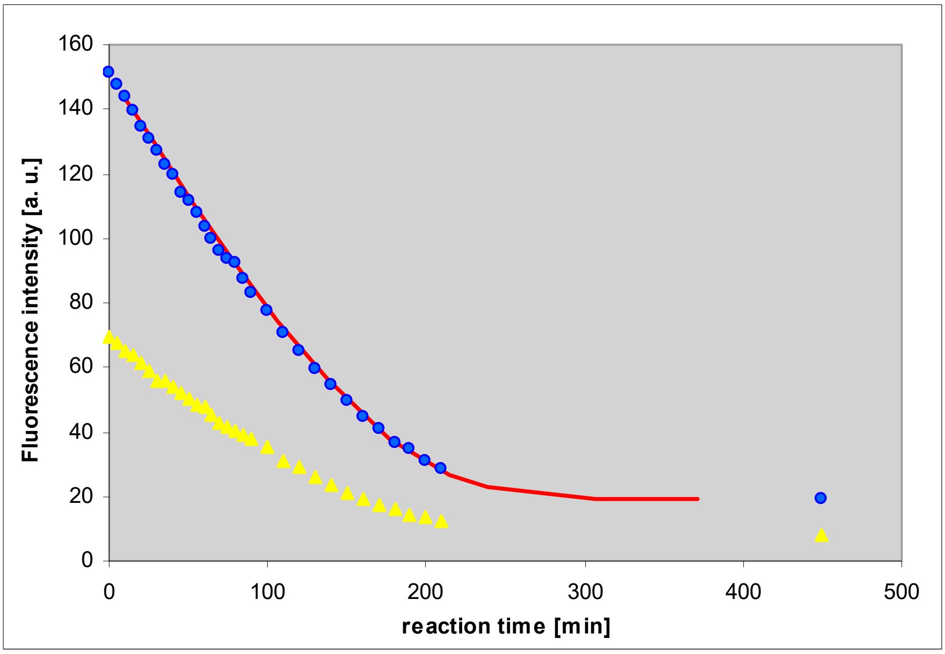

Figure 1. Time-dependence of fluorescence intensity (blue: at 400 nm; yellow: at 460 nm) measured during the ribosylation of *N*^{2},3-etheno-*O*^{6}-methylguanine (35 µM) with R1P (0.5 mM) as a ribosyl

donor, with *E. coli* PNP as a catalyst., at pH 7.3 and temperature 25° C. In red color, solid line: a theoretically calculated progress curve, assuming K_m = 7 μM and Michaelis'-Menten kinetics. Fluorescence excitation was at 290 nm.

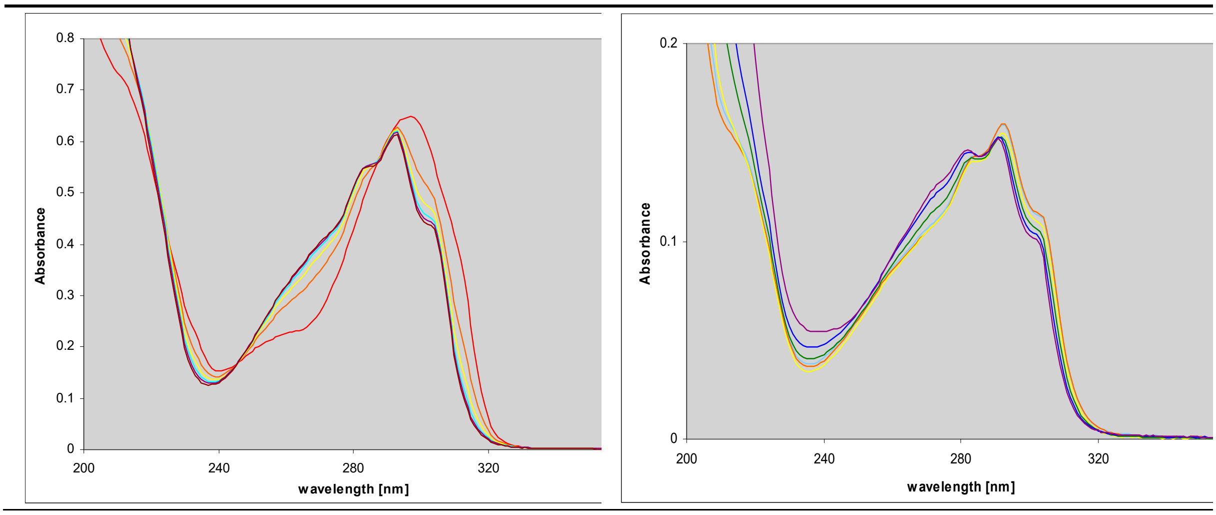

Figure 2. Spectrophotometric titrations of 1,*N*[6]-etheno-isoguanine. Left: determination of the lower (basic) pKa value: pH values from 2.9 (red) to 5.5 (violet); Right: determination of the upper (acidic) pK value: pH from 6.25 (red) to 11.5 (violet). The fitted pK_a values: 3.5 and 8.1 (±0.2).

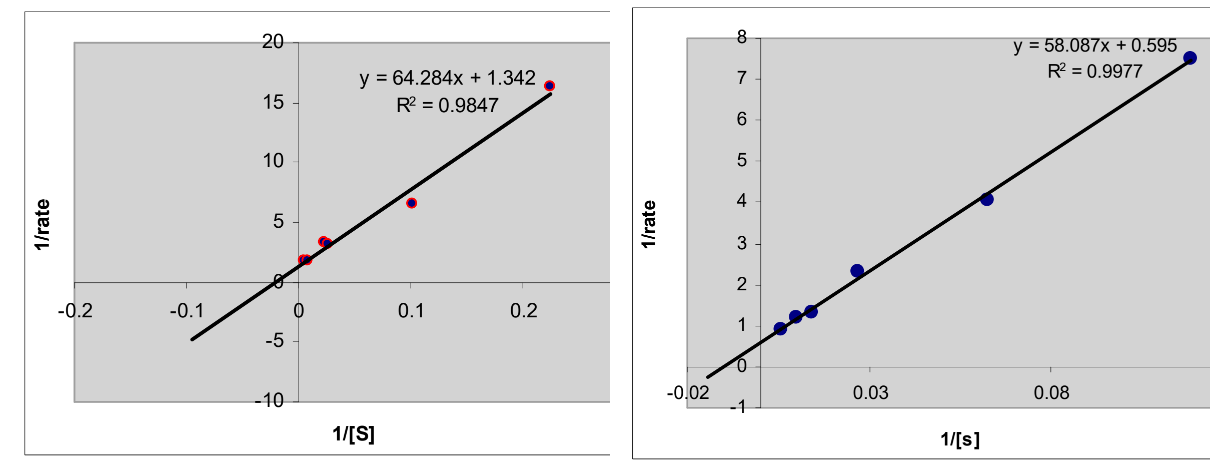

Figure S3. Double-reciprocal (Michaelis'-Menten) plots for ribosylation of 1,*N*[2]-ethenoguanine (right) and 1,*N*[6]-ethenoisoguanine (right), catalyzed by the *E. coli* PNP, wild-type. The obtained values of K_m were 48 μM and 98 μM, respectively. Conditions: 50 mM HEPS buffer, pH 7.3, with R1P (0.5 mM) as a ribosyl donor, temperature 25° C.

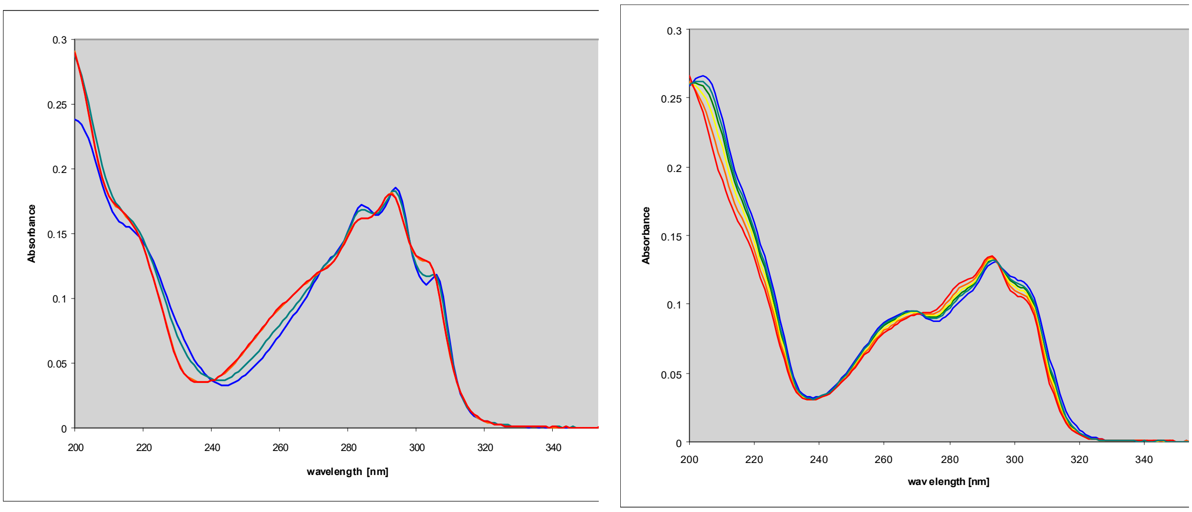

Figure 4. Phosphorolysis of *N*7- (left) and *N*9-β-D- (right) ribosides of 1,*N*[6]-etheno-isoguanosine in 50 mM phosphate buffer, pH 6.5, at 25° C, catalyzed by the *E. coli* PNP.
